# Stage-dependent effects of intermittent hypoxia influence the outcome of hippocampal adult neurogenesis

**DOI:** 10.1038/s41598-021-85357-5

**Published:** 2021-03-16

**Authors:** Maggie A. Khuu, Thara Nallamothu, Carolina I. Castro-Rivera, Alejandra Arias-Cavieres, Caroline C. Szujewski, Alfredo J. Garcia III

**Affiliations:** 1 Institute for Integrative Physiology, Section of Emergency Medicine, The University of Chicago, 5841 S Maryland Ave, Chicago, IL 60637 USA; 2 Committee On Neurobiology, The University of Chicago, Chicago, IL 60307 USA; 3 Grossman Institute for Neuroscience, Quantitative Biology and Human Behavior, The University of Chicago, Chicago, IL 60637 USA

**Keywords:** Stress and resilience, Adult neurogenesis, Experimental models of disease

## Abstract

Over one billion adults worldwide are estimated to suffer from sleep apnea, a condition with wide-reaching effects on brain health. Sleep apnea causes cognitive decline and is a risk factor for neurodegenerative conditions such as Alzheimer’s disease. Rodents exposed to intermittent hypoxia (IH), a hallmark of sleep apnea, exhibit spatial memory deficits associated with impaired hippocampal neurophysiology and dysregulated adult neurogenesis. We demonstrate that IH creates a pro-oxidant condition that reduces the Tbr2^+^ neural progenitor pool early in the process, while also suppressing terminal differentiation of adult born neurons during late adult neurogenesis. We further show that IH-dependent cell-autonomous hypoxia inducible factor 1-alpha (HIF1a) signaling is activated in early neuroprogenitors and enhances the generation of adult born neurons upon termination of IH. Our findings indicate that oscillations in oxygen homeostasis, such as those found in sleep apnea, have complex stage-dependent influence over hippocampal adult neurogenesis.

## Introduction

Sleep apnea is a common clinical condition estimated to afflict over a billion individuals throughout the world^1^. Along with posing as a risk factor for negative cardiorespiratory conditions^2–5^, sleep apnea also increases the risk for cognitive impairment^6,7^. Although more commonly associated with the aging population^8–11^, children and adolescents may also suffer from sleep apnea and its neurocognitive consequences^6,12,13^. Experimental paradigms of IH that model sleep apnea cause deficits to hippocampal-based learning and memory^14–16^ through impairments to hippocampal neurophysiology^17–22^.

In addition to supporting synaptic and intrinsic neuronal properties in the hippocampus, hippocampal adult neurogenesis appears to be important for cognitive performance^23–26^ and behavior during stress and depression^27,28^. Immature neurons derived from this process have distinctly different intrinsic and circuit properties when compared to the more mature counterparts of the circuit^29–31^.

Neural precursors experience two critical periods during adult neurogenesis^32,33^. The first critical period occurs early in development, during the transition of T-box brain protein 2 (Tbr2) expressing intermediate neural progenitors (INPs) to neuroblasts^32^. INPs typically express Tbr2 within the first three days of birth dating, however, by seven days post birth dating, these cells begin to transition and also express the neuroblast marker, doublecortin (DCX)^34^. Surviving INPs that successfully transition into neuroblasts must then undergo a second critical period when the neuroblast transitions to an immature granule neuron^33^. By the third week of development neuroprogenitors can be identified as DCX^+^ no longer express Tbr2^34^. These surviving immature granule neurons incorporate into the pre-existing network of the granule layer in the dentate gyrus and persist for multiple months with minimal apoptosis^33,35–37^.

Paradigms of IH used to model sleep apnea report that thirty or more days of IH enhances the neural precursor pool^18,38^ while also suppressing the generation of adult-born neurons^18^. Exposure to IH can promote a pro-oxidant state (i.e., increased oxidative stress) and has differential effects on early neural precursors and adult-born neurons^18^. These differences raise the question of whether IH experienced during different stages of adult neurogenesis have distinct outcomes on the generation of adult-born neurons. We sought to address this by investigating how targeted IH exposure during (1) the first critical period (IH_EARLY_); and (2) the second critical period (IH_LATE_), impacted the generation of adult born neurons. We found that IH_EARLY_ increased the proportion of birth-dated neurons; whereas, IH_LATE_ reduced the proportion of birth-dated neurons. Our findings also indicate that an increase of adult-born neurons caused by IH_EARLY_ involves cell-autonomous HIF1a activity while suppression of adult neurogenesis by IH_LATE_ is uninfluenced by such activity. These observations suggest that IH-dependent cell-autonomous HIF1a signaling acts to promote neuronal fate during the first critical period, but does not mitigate the reduction of adult born neurons if IH is experienced during the second critical period of adult neurogenesis.

## Results

### A stage related role for IH to regulate adult neurogenesis

**Figure 1:**
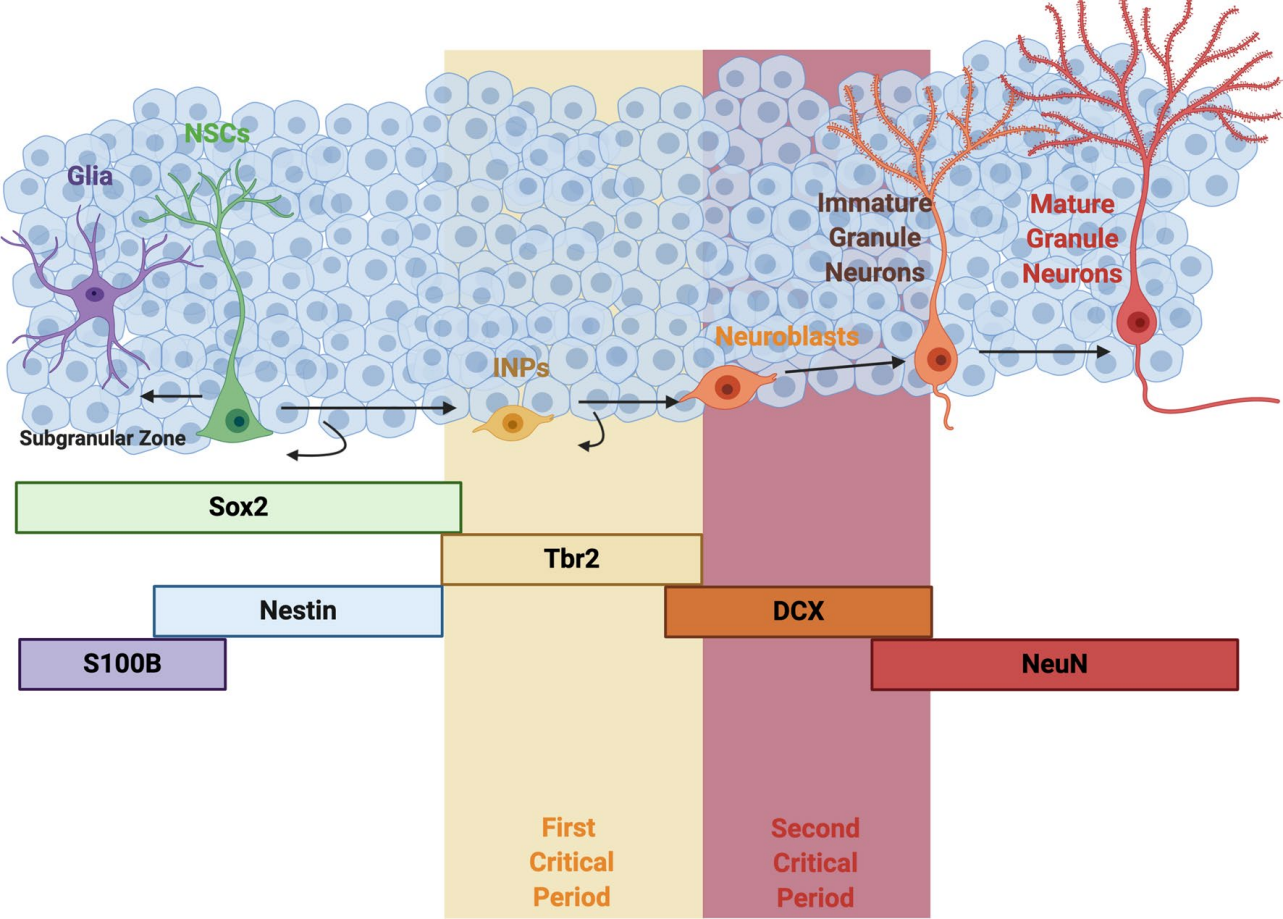
Developmental trajectory and associated markers of hippocampal adult neurogenesis. The developmental trajectory of NSCs within the SGZ is shown. One cycle of neurogenesis, encompassing the transition from neuroprogenitor to neuron, occurs approximately over 30 days. Key developmental stages and associated markers are delineated. Highlighted by the yellow band, the first critical period predominantly encompasses the population of cells transitioning to the neuroblast stage while the second critical period, highlighted in red, encompasses the transition of neuroblasts to immature granule neurons.

**Figure 2:**
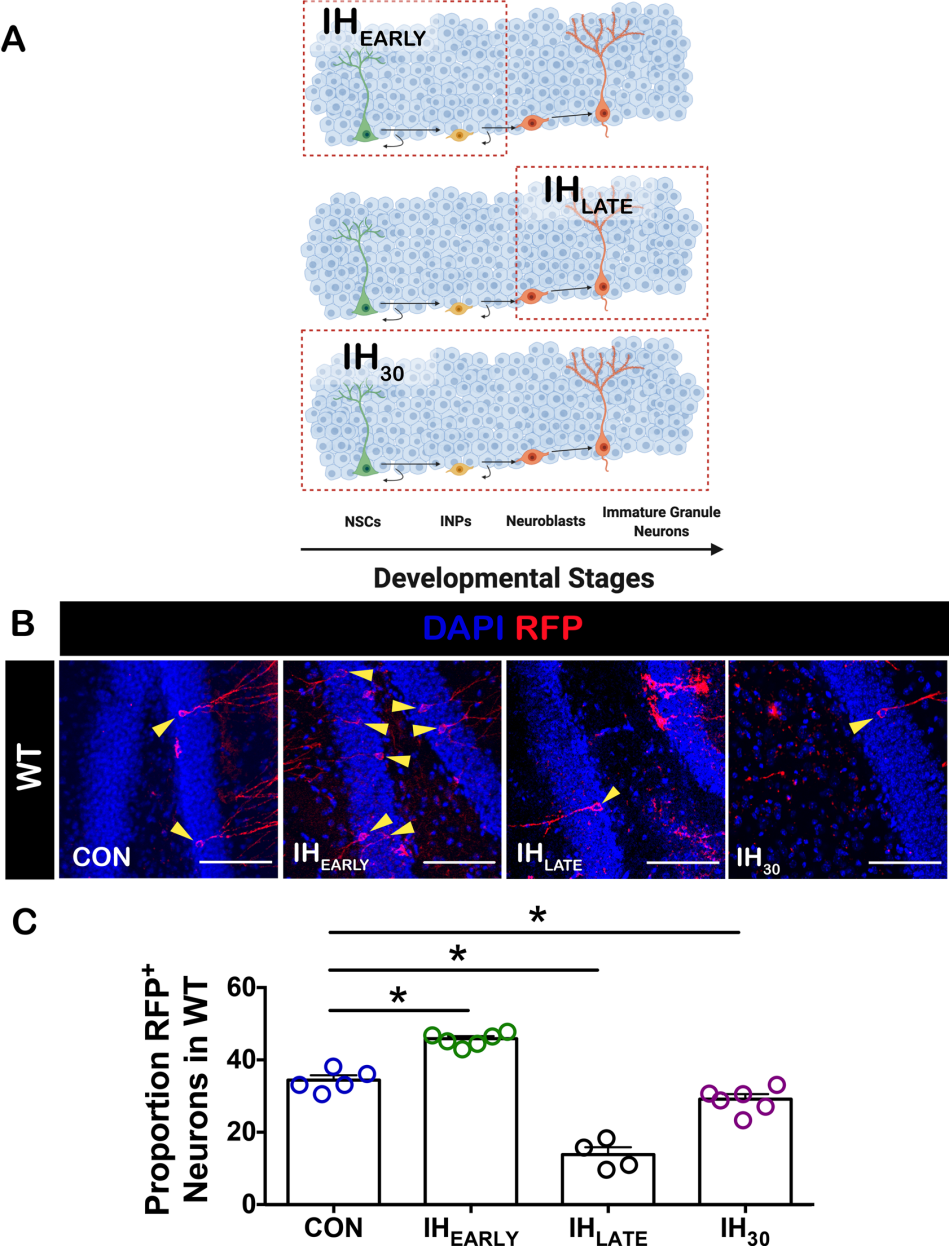
Stage-dependent effects of IH are revealed when neural precursors are exposed to IH_10_ during different stages of adult neurogenesis. (**A**) A diagram illustrates the three different birth dating paradigms employed in this study: Labeled Nestin^+^ NSCs experienced exposure to ten days of IH during the first critical period of neurogenesis followed by a recovery period of 20 days prior to sacrifice (IH_EARLY_; Top), exposure to ten days of IH during the second critical period (IH_LATE_; Middle), or exposure to IH for 30 days, encompassing both critical periods (IH_30_; Bottom). Prior to any exposure to IH, all animals were injected with tamoxifen. The red outlines in each of the paradigms highlight the approximate developmental stages during which birth-dated cells were directly exposed to IH. (**B**) Representative images for RFP^+^ neurons derived from wildtype (WT) birth-dated NSCs from CON, IH_EARLY_, IH_LATE_, and IH_30_ conditions were examined. Yellow triangles identify RFP^+^ neurons. Scale bars are 100um. (**C**) Differences were observed between the proportion of RFP^+^ neurons derived from WT NSCs in all conditions (CON: n = 5, IH_EARLY_: n = 6, IH_LATE_: n = 4, IH_30_: n = 6). One way ANOVA with Dunnet’s multiple comparisons: P =  < 0.0001, F = 91.05. CON vs. IH_EARLY_: P < 0.0001, CI of diff = -16.21 to -6.664. CON vs. IH_LATE_: P < 0.001, CI of diff = 15.34 to 25.92. CON vs. IH_30_: P < 0.05, CI of diff = 0.5268 to 10.07).

Differentiation from hippocampal neural stem cell to immature granule neuron is a multistep process that occurs approximately over thirty days^39–42^ (Fig. 1). During this process, neural precursors in the subgranular zone of the dentate gyrus (SGZ) must undergo two distinct critical periods of neurogenesis. To resolve how IH exposure during these critical periods influenced the generation of adult-born neurons, we birth dated a cohort of Nestin^+^ NSCs and tracked their development using different IH exposure protocols over the course of thirty days. In IH_EARLY_, animals with labeled Nestin^+^ NSCs were exposed to ten days of IH immediately following the 1 d.p.i. of tamoxifen. After IH, the animals were allowed twenty days to recover in room air where labeled neuroprogenitors were allowed to continue to develop before sacrifice (Fig. 2A, top). In IH_LATE_, labeled Nestin^+^ NSCs were allowed to develop for 20 d.p.i. in room air prior to ten days of IH exposure and were sacrificed immediately following the conclusion of IH (Fig. 2A, middle). IH_30_ cohorts experienced IH during the full thirty-day period (Fig. 2A, bottom). Thus IH_EARLY,_ encompassed the initial critical period; IH_LATE_ overlapped with the second critical period, and IH_30_ encompassed both critical periods.

By birth dating a cohort of Nestin^+^ NSCs, we were able to follow the fate of these cells to discern the specific consequences of IH exposure during these critical periods. When compared to control (CON), the proportion of birth-dated neurons was increased following IH_EARLY_ (Fig. 2C), yet was decreased after IH_LATE_ (Fig. 2C) and IH_30_ (Fig. 2C). These findings revealed that if IH is experienced during the first critical period of adult neurogenesis and recovery is allowed, neurogenesis is enhanced. However, when IH is experienced later during the second critical period, the generation of neurons is negatively regulated, similar to the effect of IH when experienced over an entire cycle of the process (i.e., IH_30_).

### Early neural precursors are differentially affected by IH_10_

**Figure 3:**
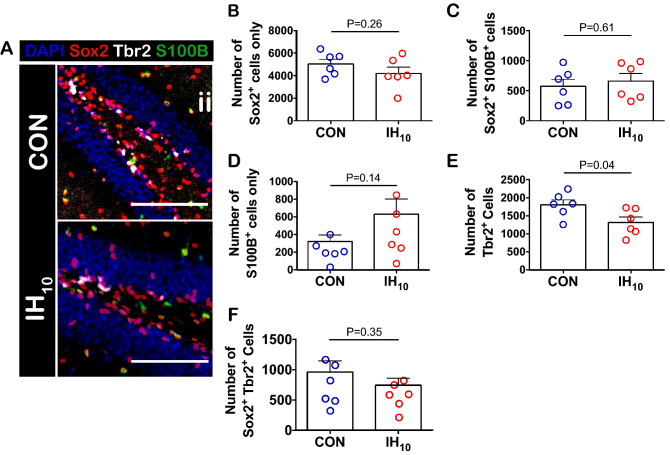
IH_10_ suppresses the number of Tbr2^+^ cells in the SGZ. (**A**) Representative images of the SGZ from CON and IH_10_ treated animals. Scale bars are 100 μm. (**B**–**D**) There were no differences in the number of pluripotent Sox2^+^ NSCs (CON: n = 6, IH_10_: n = 6), quiescent Sox2^+^/S100B^+^ co-labeled NSCs (CON: n = 6, IH_10_: n = 6), or S100B^+^ astrocytes (CON: n = 6, IH_10_: n = 6). (**E**) The number of Tbr2^+^ INPs was decreased following IH_10_ (CON: n = 6, IH_10_: n = 6). (**F**) There was no difference in the transition from NSCs to INPs in Sox2^+^/Tbr2^+^ cells (CON: n = 6, IH_10_: n = 6).

**Figure 4:**
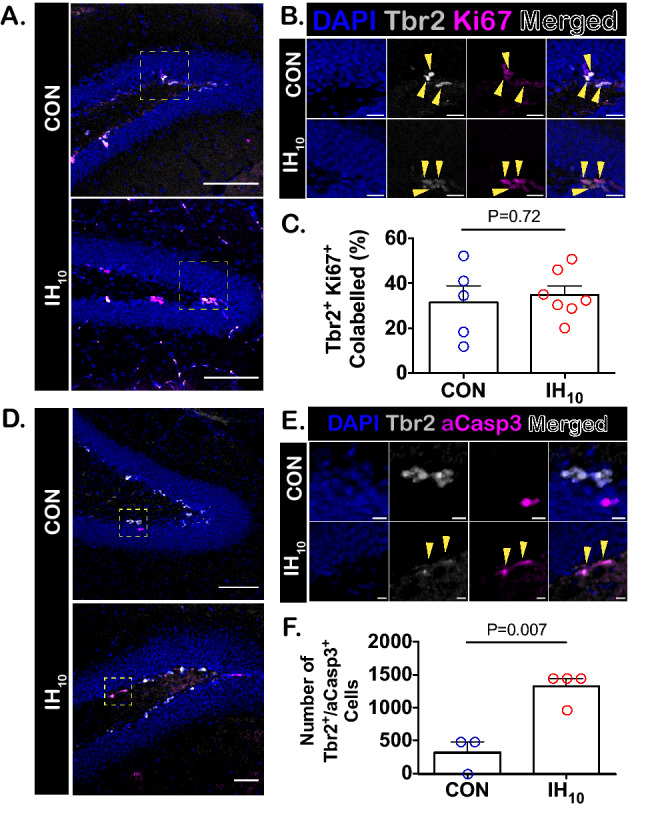
While IH_10_ does not enhance proliferation among Tbr2^+^ cells, activated caspase-3 expression is enhanced among Tbr2^+^ cells by IH_10_. (**A**) Representative images of the dentate gyrus from CON and IH_10_ show similar labeling for Ki67^+^ and Tbr2^+^ cells (CON: n = 5, IH_10_: n = 7). Scale bars are 100 μm. (**B**) Magnified images from yellow dashed boxes in (**A**) from CON and IH_10_. Yellow triangles denote co-labelling among cells. Scale bars for inset images are 20 μm. **C.** No difference between co-labeling of Tbr2^+^/Ki67^+^ cells suggesting IH_10_ does not impact proliferative activity among Tbr2^+^ cells. (**D**) Representative images of the dentate gyrus from CON and IH_10_ treated animals showing the labeling for activated capase-3^+^ and Tbr2^+^ cells (CON: n = 3, IH_10_: n = 4). (**E**) Magnified images from yellow dashed boxes in (**D**) from CON and IH_10_. Yellow triangles denote co-labeling among cells. Scale bars for inset images are 20 μm. **F.** Co-labeling of Tbr2^+^/activated capsase-3^+^ cells is greater in CON compared to IH_10_ (P = 0.007) indicating that IH_10_ activates apoptosis among Tbr2^+^ cells.

To determine how IH_EARLY_ stimulates the generation of neurons and how this was related to the enhancement of early neuroprogenitor pools as previously described^18^, we examined how an abbreviated ten-day exposure to IH with no recovery time (IH_10_) specifically impacted different early neural precursor pools. The total number of pluripotent NSCs (Sox2^+^ cells), quiescent NSCs (Sox2^+^ S100B^+^ cells)^43,44^, astrocytes (S100B^+^ cells), and INPs (Tbr2^+^ cells) were assessed in SGZ immediately following IH_10_ (Fig. 3A). IH_10_ did not change the number of pluripotent NSCs (Fig. 3B), quiescent NSCs (Fig. 3C) or astrocytes (Fig. 3D) suggesting that 10 days of IH is not sufficient to activate signaling that enhances NSC proliferation as seen following a longer duration of IH^18,38^. However, IH_10_ was sufficient in reducing the number of Tbr2^+^ cells (Fig. 3E). Cellular co-localization of Tbr2 and Sox2 represents a transitional state from NSC to INP^45^. IH did not change in the number of cells co-expressing Tbr2 and Sox2 (Fig. 3F). Additionally, proliferative activity in Sox2^+^ cells and Tbr2^+^ cells was assessed by KI67 co-localization in the two cell types. Neither the proportion of proliferating Sox2^+^ NSCs (Supplemental Figure S1) nor the proportion of proliferating Tbr2^+^ cells (Fig. 4A–C) were affected by IH_10_. As the decrease in Tbr2^+^ cells did not appear to be related to reduced proliferative activity in the NSC population nor the Tbr2^+^ cell population, we sought to examine how IH_10_ influenced cell death among Tbr2^+^ cells by examining co-localization with activated caspase-3. Following exposure to IH_10_, the number of Tbr2^+^ cell co-labeled with activated caspase-3 increased (Fig. 4D–F). Thus, the primary cause for the reduction in the pool of Tbr2^+^ cells appeared to be driven by an IH-dependent increase in cell death among INPs. These findings demonstrated that without the twenty day recovery allowed occurring with IH_EARLY_, the IH_10_ suppresses the Tbr2 + cell population and thus, suggests that IH initializes two seemingly opposing processes on adult neurogenesis prior to transitioning from the first critical period.

### The pro-oxidant state caused by IH_10_ increases HIF1a co-localization in Tbr2^+^ INPs

A number of studies have reported that IH causes a pro-oxidant state that can promote oxidative stress^22,46,47^, which could lead to oxidative injury and cell death. Therefore, we measured malondialdehyde (MDA) content via thiobarbituric acid reactive substances (TBARS) assay to assess the state of lipid peroxidation as a measure for a shift in redox state toward pro-oxidant conditions that could cause oxidative stress.

**Figure 5:**
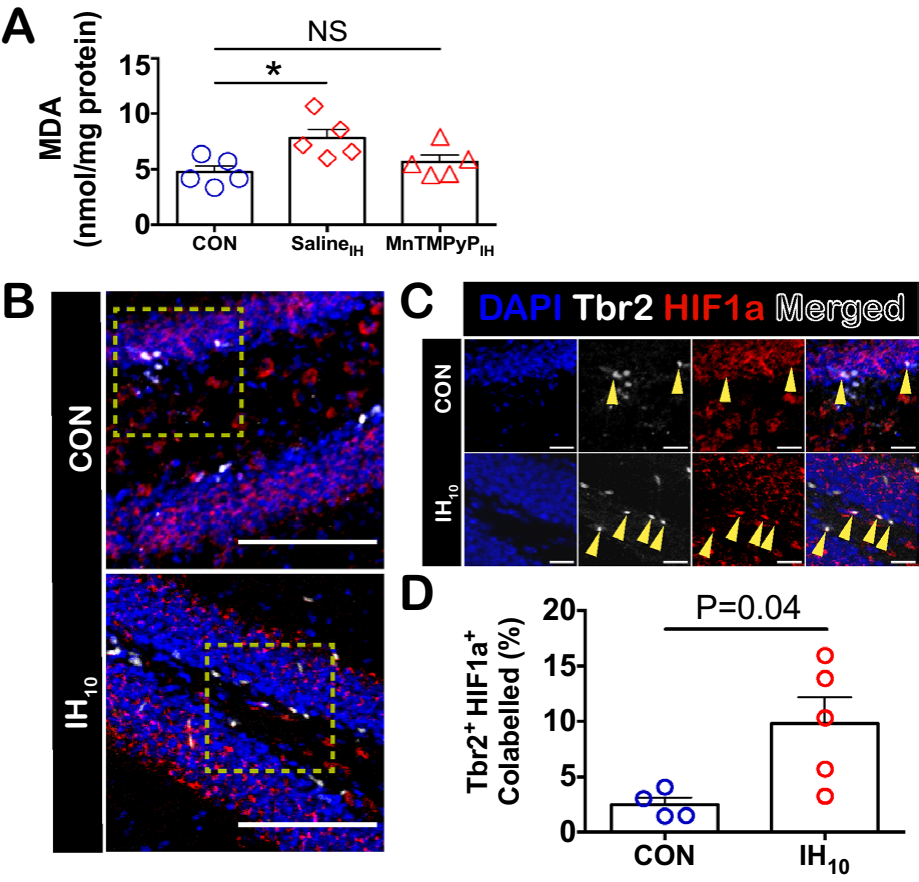
IH_10_ increases hippocampal oxidative stress and HIF1a expression in Tbr2^+^ cells. (**A**) Following exposure to IH, oxidative stress as quantified by MDA content was increased in IH_10_ animals (Saline_IH_, red diamonds). In contrast, MDA content in IH_10_ animals that received concurrent MnTMPyP treatment (MnTMPyP_IH_, red triangles) was not significantly different when compared to control animals (CON: n = 5, Saline_IH_: n = 5, MnTMPyP_IH_: n = 5). One-way ANOVA with Dunnett’s multiple comparisons: P = 0.0232, F = 5.234. CON vs Saline_IH_: P < 0.05, Cl of diff = − 5.455 to − 0.6288. CON vs MnTMPyP_IH_: P > 0.05, Cl of diff = − 3.334 to 1.493 (**B**). Representative images for CON and IH_10_ are shown. Scale bars are 100 μm. Yellow boxes highlight the inset images. (**C**) Inset images are shown. Yellow triangles indicate examples of co-labeled cells for CON and IH_10_. Scale bars for inset images are 20 μm. (**D**) An increased proportion of HIF1a^+^ Tbr2^+^ INPs was observed following IH_10_ (CON: n = 4, IH_10_: n = 5).

TBARS assays were performed in hippocampal homogenates from control (n = 5), IH_10_ (Saline_IH_, n = 5), and IH_10_ with concurrent administration of the superoxide anion scavenger, MnTMPYP, (MnTMPyP_IH_, n = 5). While hippocampal MDA content increased in Saline_IH_, MDA content in MnTMPyP_IH_ was similar to levels found in control (Fig. 5A). These findings indicate that IH causes a shift toward a pro-oxidant state that increases hippocampal lipid peroxidation and is prevented by antioxidant treatment. Since enhanced HIF1a signaling in the hippocampus has been shown to contribute to the pro-oxidant condition caused by IH^22^ and Tbr2^+^ INPs are susceptible to oxidative stress^34^, we next examined how IH affected HIF1a expression among early neural precursors and the potential role that an IH-dependent pro-oxidant state has on Tbr2^+^ cells.

**Figure 6:**
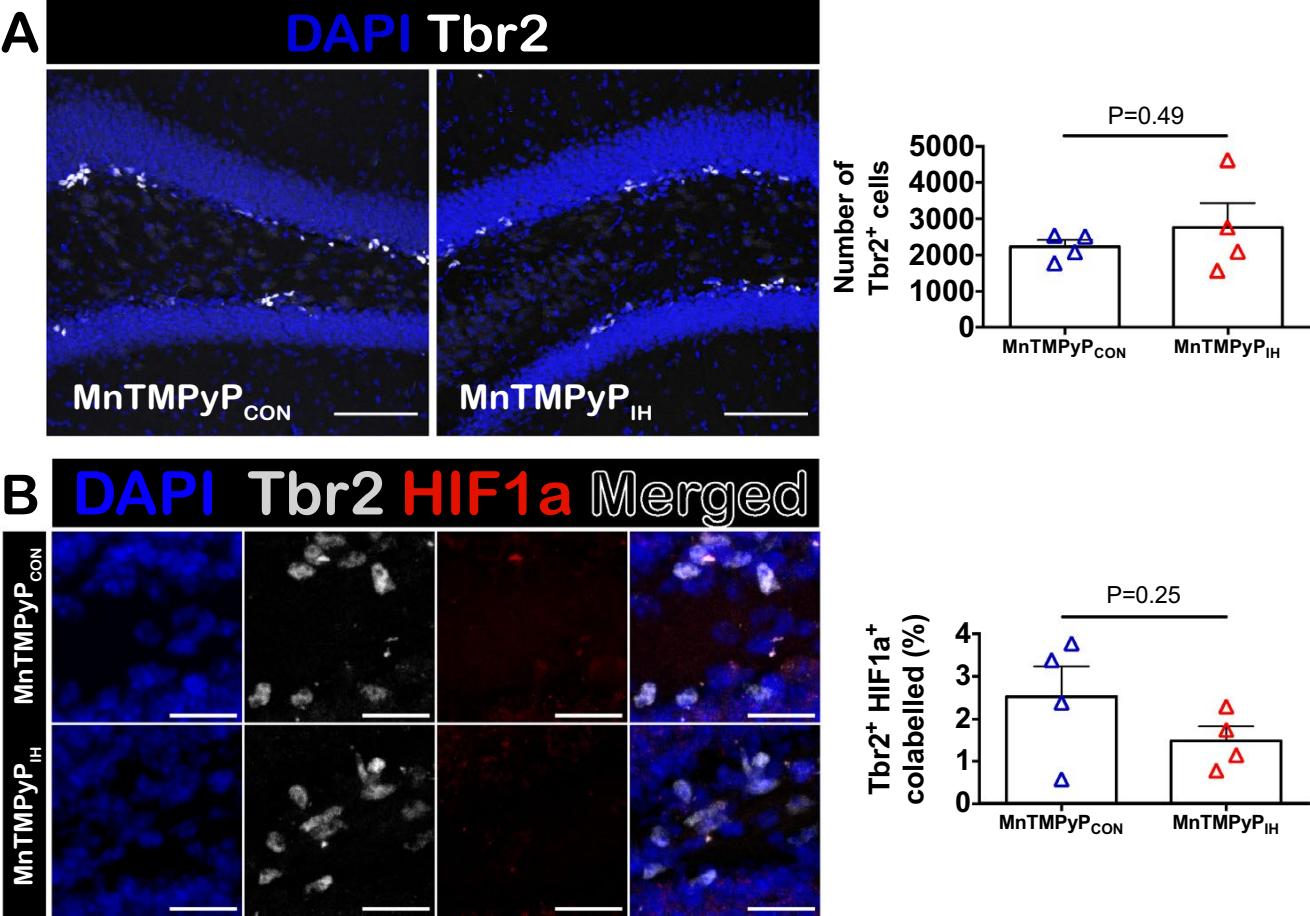
Antioxidant treatment during IH_10_ prevents a reduction in Tbr2^+^ cells and prevents an increase in HIF1a expression. (**A**) Representative images of SGZ in MnTMPyP-treated control (MnTMPyP_CON_) and MnTMPyP-treated IH_10_ animals (MnTMPyP_IH_). Scale bars are 100 μm. There was no difference in the number of Tbr2^+^ INPs (CON: n = 4, IH_10_: n = 4). (**B**) Representative images of co-labeled HIF1a^+^ Tbr2^+^ cells. Scale bars are 20 μm. The proportion of HIF1a^+^ Tbr2^+^ was also not different following IH_10_ with MnTMPyP (MnTMPyP_CON_: n = 4, MnTMPyP_IH_: n = 4). Scale bars are 20 μm.

The proportion of HIF1a^+^ Sox2^+^ pluripotent NSCs was unchanged by IH (Supplemental Figure S2) in the SGZ, but the proportion of HIF1a^+^ Tbr2^+^ co-labeled INPs increased following IH (Fig. 5B–D). Further analysis also revealed a difference in variance in HIF1a^+^ Tbr2^+^ co-labeled INPs (Fig. 5D,F(4,3) = 17.57, P = 0.04) suggesting that IH-dependent HIF1a signaling in the Tbr2^+^ INPs may be dependent on the immediate redox environment surrounding these cells. To determine whether the pro-oxidant state prevented the reduction in Tbr2^+^ INPs, and effects of HIF1a expression in these cells, we examined Tbr2 and HIF1a co-localization in the SGZ of animals treated with MnTMPyP. Treatment with MnTMPyP during IH_10_, mitigated the reduction of Tbr2^+^ INPs (Fig. 6A) and prevented the increase of HIF1a and Tbr2 colocalization in INPs (Fig. 6B). Moreover, a difference in variance in HIF1a^+^ Tbr2^+^ INPs was no longer present between the groups (Fig. 6B, F(3,3) = 4.593, P = 0.242). These observations indicate that the pro-oxidant state produced by IH suppresses the pool of INPs and specifically upregulates HIF1a in Tbr2^+^ cells.

### Hemizygosity in HIF1a prevents enhanced neurogenesis following IH_EARLY_ but not IH_LATE_

**Figure 7:**
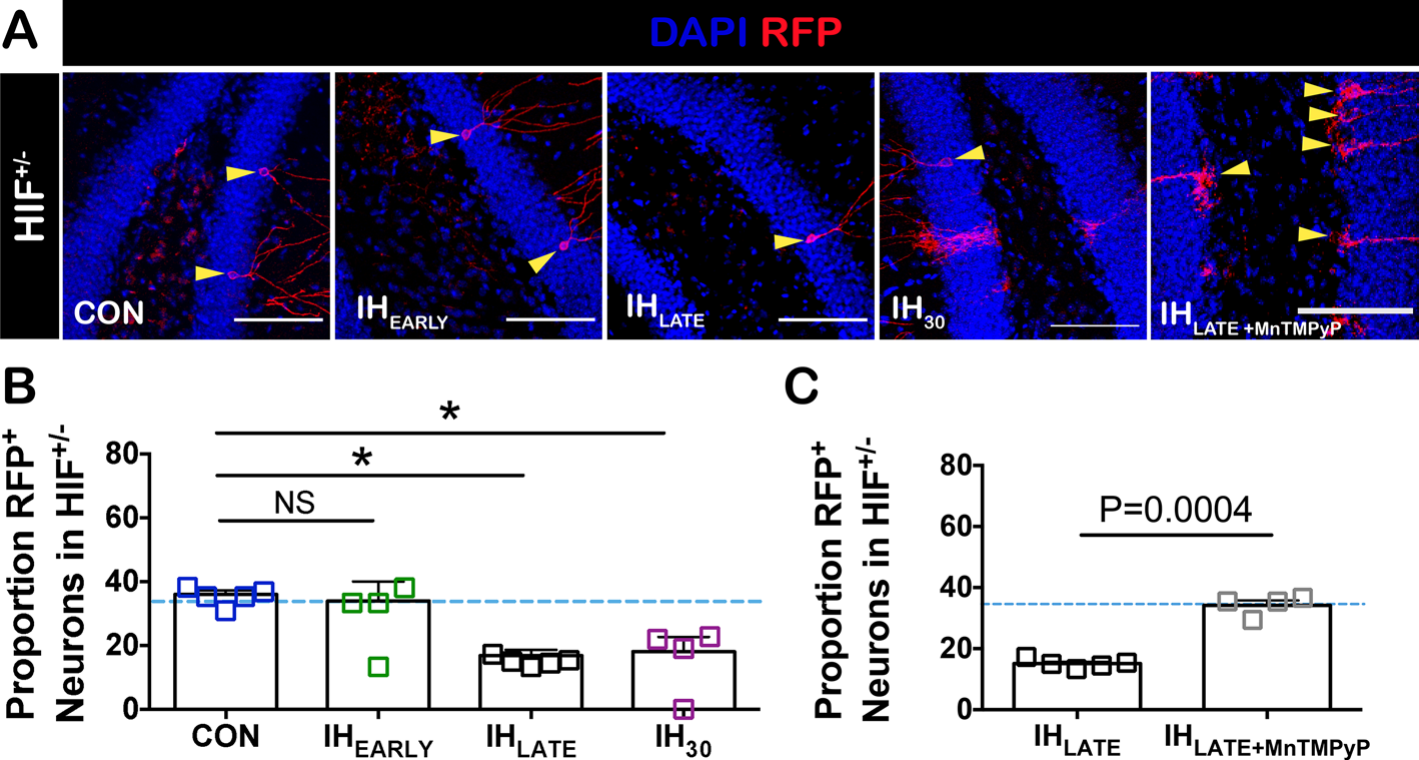
Stage-dependent differences in cell-autonomous HIF1a signaling among neural progenitors is revealed by HIF1a hemizygosity and antioxidant treatment. (**A**) Representative images from CON, IH_EARLY_, IH_LATE_, and IH_30_ for RFP^+^ labeled neurons derived from HIF1a^+/−^ birth dated NSCs. Yellow triangles denote RFP^+^ neurons. Scale bars are 100 μm. (**B**) While no difference between the proportion of RFP^+^ neurons in CON and IH_EARLY_ (CON: n = 5, IH_EARLY_: n = 4) was observed, differences were detected between CON and IH_LATE_ (IH_LATE_: n = 5) and CON and IH_30_ (IH_30_: n = 4). The percentage of RFP^+^ neurons derived from HIF1a^+/−^ NSCs (blue squares) was similar to that of neurons generated from birth dated WT NSCs (light blue dashed line, from Fig. 2 CON). * = P < 0.05; NS = P ≥ 0.05. one-way ANOVA with Dunnet’s multiple comparisons: P = 0.0015, F = 8.856. Con vs. IH_EARLY_: P > 0.05 (NS), CI of diff = − 7.101 to − 18.71. Con vs. IH_LATE_: P < 0.01, CI of diff = 8.067 to 32.40. Con vs. IH_30_: P < 0.01, CI of diff = 6.492 to 32.30. (**C**) Concurrent treatment with MnTMPyP during IH_LATE_ prevented the reduction in the proportion of RFP^+^ neurons derived from HIF1a^+/-^ NSCs (n = 4, grey squares) when compared to IH_LATE_ (replotted from **B**). Light blue dashed line represents proportion of neurons generated from birth dated WT NSCs (from Fig. 2 CON).

While hippocampal HIF1a appears to drive ROS production by IH^22^, cell-autonomous HIF1a signaling in neural precursors can also serve as a pro-neurogenesis factor^34,48,49^. Thus, the enhanced neurogenesis observed with IH_EARLY_ could be mediated by upregulation of HIF1a in INPs, promoting the increase in the proportion of adult born neurons after the selective pressure of IH is removed. To test this, we subjected mice hemizygous for HIF1a (HIF1a^+/-^) among birth-dated Nestin^+^ NSCs to IH_EARLY_, IH_LATE,_ and IH_30_ (Fig. 7A). The proportion of birth-dated neurons produced from HIF1a^+/−^ NSCs was unchanged by IH_EARLY_ (Fig. 7B) suggesting that the enhanced generation of neurons caused by IH_EARLY_ in WT NSCs involves cell-autonomous HIF1a signaling during the first critical period of adult neurogenesis. However, IH_LATE_ and IH_30_ both reduced the proportion of neurons derived from HIF1a^+/−^ NSCs (Fig. 7B). To test whether an IH-dependent pro-oxidant state played a role in suppressing adult neurogenesis during the second critical period, we administered MnTMPyP concurrent with IH_LATE_ in mice hemizygous for HIF-1a among birth-dated INPs. When compared to IH_LATE_ untreated mice, the proportion of adult-born neurons derived from HIF1a^+/−^ neuroprogenitors was greater with concurrent MnTMPyP treatment and was also similar to the mean proportion of new neurons generated in control mice (Fig. 7C). Thus, the proportion of adult born neurons was rescued by MnTMPyP administration but not by HIF-1a hemizygosity during IH_LATE_, suggesting that the IH-dependent suppression of neurogenesis observed during the second critical period is independent of HIF1a.

## Discussion

The continued production of new hippocampal neurons throughout adulthood is a multistage process sensitive to the state of oxygenation^34,50–52^. In contrast to the improvement of neurogenesis using intermittent hypoxia designed for therapeutic purposes^53–56^, the paradigm of IH used in this study reflects the fast-recurrent oscillations in oxygen homeostasis that is associated with sleep apnea. Previous work using IH as a model for sleep apnea demonstrated that 30 days of exposure to IH produces two distinct effects on hippocampal adult neurogenesis. Thirty or more days of IH increases the number and proliferation of early neural progenitors^18^, yet even with enhanced proliferative activity, IH_30_ also causes a net reduction in the number of adult born neurons^18,38^. By following the development of discretely labeled neural progenitors exposed to ten days of IH at different stages of the process (i.e., IH_EARLY_ and IH_LATE_), as well as examining the immediate effect of ten days of IH on entire populations of early progenitors (IH_10_), we demonstrate that IH has stage-related influence over adult neurogenesis. We show that IH-dependent pro-oxidant state negatively regulates Tbr2 + neuroprogenitors and suppresses the neuronal fate derived from NSCs. Our experiments also demonstrate the potential for IH to initialize pro-neurogenesis signaling that involves cell-autonomous HIF1a activity. This pro-neurogenesis signaling appears to be initialized in early neuroprogenitors to promote the neuronal fate. Together our results suggest that impact of IH on the generation of adult-born neurons is determined by the intersection during different and antagonizing processes initiated by IH.

Cells involved in adult neurogenesis must pass through two distinct critical periods where either progression toward neuronal differentiation occurs or the process is aborted via programmed cell death^32,33,36,37^. Our IH_EARLY_ and IH_LATE_ protocols dissected how IH influenced the generation of adult-born neurons when experienced during the two different critical periods of adult neurogenesis. Consistent with the suppressive action of IH_30_ on the generation of adult born neurons, IH_LATE_ suppressed the proportion of adult born neurons derived from birth dated neural precursors. In contrast to IH_LATE_, the IH_EARLY_ protocol enhanced the proportion of adult-born neurons relative to other cell types. This effect of IH_EARLY_ may have been related to the IH-mediated enhancement of proliferation and number of the NSC population as previously reported with thirty or more days of IH^18,38^. Exposure to IH_10_ did not cause an increase in proliferation nor number of early neural precursors (Supplemental Figure S1 and Fig. 3). Additionally, the number of Sox2^+^ Tbr2^+^ cells was also unchanged suggesting that the efficiency of transition of NSCs to INPs was unaffected by IH_10_. Thus, our findings indicate that unlike IH_30_, the abbreviated duration of IH_10_ was not sufficient at driving a measurable increase either in number or in the proliferative activity of the early neural precursor populations.

IH_10_ unexpectedly reduced the number of Tbr2^+^ INPs. This reduction in Tbr2^+^ INPs coincided with increased co-localization of activated caspase-3 indicating that IH negatively regulates INPs via programmed cell-death during the first critical period of adult neurogenesis. As adult neurogenesis is a finite process known to rapidly drop off with age^57–60^, these findings demonstrate the potential for IH to also negatively impact early stages of hippocampal adult neurogenesis. While it may seem to contradict previous reports demonstrating the ability for IH to expand the early progenitor populations^18,38^, it is important to recognize that these previous studies used thirty or more days of IH. While our experiments illustrate the ability of IH to negatively regulate Tbr2^+^ INPs, our data suggest that IH exposure with durations greater than ten days is required to see evidence of pro-neurogenesis signaling within early neuronal progenitor populations.

The IH-dependent reduction of Tbr2^+^ INPs also coincided with increased hippocampal lipid peroxidation; indicating a shift toward a pro-oxidant state capable of causing oxidative stress. Such oxidative stress could drive the observed increase in programmed cell death among INPs. Indeed, MnTMPyP administration prevented the reduction of Tbr2^+^ cell number by IH_10_. Systemic hemizygosity of HIF1a prevents the IH-dependent pro-oxidant state^22^ indicating that, in general, HIF1a is a primary contributor to hippocampal oxidative stress caused by IH. We observed that IH increased HIF1a co-localization among Tbr2^+^ INPs. Although this finding was consistent with the perspective that cell-autonomous HIF1a in Tbr2^+^ INPs caused oxidative stress among neural progenitors, HIF1a is also normally present in multiple cell types throughout the SGZ where it acts as a pro-neurogenesis signal among early neuroprogenitors^48,61^.

Our experiments using labeled nestin-positive HIF1a^+/−^ neural progenitors allowed us to examine how cell-autonomous hemizygosity of HIF1a influenced the generation of labeled adult-born neurons under control conditions and different IH protocols. While genetic ablation of both copies of HIF1a in NSCs suppresses the generation of adult-born neurons^61^, HIF1a^+/−^ NSCs produced similar proportions of neurons when compared to that produced from wildtype NSCs. This observation indicates that a single copy of HIF1a sufficiently supports adult neurogenesis and that HIF1a hemizygosity does not influence the efficacy to generate hippocampal adult-born neurons under normal circumstances. The proportion of adult-born neurons derived from HIF1a^+/−^ NSCs was reduced in both IH_LATE_ and IH_30_. These phenomena were consistent with our observations using wildtype NSCs in the same protocols. However, the proportion of adult born neurons originating from birth-dated HIF1a^+/−^ NSCs was unaffected by IH_EARLY_. This outcome was different from the effect of IH_EARLY_ to increase the proportion of adult born neurons originating from birth-dated wildtype NSCs.

To provide better resolution into the role of NSC genotype on neuronal proportions following the different experimental protocols, we performed a reanalysis of data comparing neuronal proportions derived from wildtype (Fig. 2) and HIF1a^+/−^ (Fig. 7) NSCs (Supplemental Figure S3). In IH_EARLY_, where labeled NSCs experience IH early in adult neurogenesis, a smaller proportion of neurons was generated from HIF1a^+/−^ NSCs when compared to neurons generated from wildtype NSCs (Supplementary Figure S3B). However, in IH_LATE_, where IH is experienced after the first critical period and during the final stages of neurogenesis, the neuronal proportions generated by HIF1a^+/-^ and wildtype NSCs were similar to one another (Supplemental Figure S3C). These re-analyses show that IH dependent cell-autonomous HIF1a signaling during early adult neurogenesis favors the generation of adult-born neurons; whereas, such cell-autonomous HIF1a signaling after the first critical period does not impact how IH suppresses the generation of adult-born neurons during this period. Indeed, in IH_30_, where IH is experienced both early in adult neurogenesis and after the first critical period of the process, the proportion of neurons originating from HIF1a^+/−^ NSCs was smaller compared to that from wildtype NSCs (Supplemental Figure S3D).

Evidence for IH to initialize pro-neurogenesis signaling among early neural precursors has been previously provided using thirty or more days of IH^18,38^. Although 10 days of IH did not cause measurable increases in either proliferation or population size of unlabeled neural precursors, the demonstration that exposure to IH_EARLY_ results in larger proportions of labeled neurons derived from wildtype NSCs supports the perspective IH activates pro-neurogenesis signaling during early stages of adult neurogenesis. The differences between genotypes using IH_30_ also indicate that the pro-neurogenesis role of cell-autonomous HIF1a signaling in early neural precursors plays an important role for partly mitigating but not preventing the suppressive action of IH_30_ on the neuronal fate. Thus, these results suggest that the efficacy of IH-dependent pro-neurogenesis signaling is influenced by the duration of IH.

Although our study did not explicitly identify how IH-dependent HIF1a signaling may be promoting the neuronal fate, it is possible that IH may be increasing the proliferation of progenitors or Tbr2^+^ cells. However, this remains to be experimentally tested. Future work must also determine the downstream signaling pathway by which cell-autonomous HIF1a signaling acts to promote adult neurogenesis in response to IH. Canonical targets of HIF1a, such as vascular endothelial growth factor^62–64^ and erythropoietin^64,65^ can positively regulate adult neurogenesis and thus, may potentially facilitate and promote adult neurogenesis in response to IH.

Using IH_30_ demonstrates that when IH exposure encompasses a complete cycle of adult neurogenesis, the net impact is a reduction in adult born neurons (see^18^ and Fig. 2). Antioxidant treatment prevented this net suppression of adult-born neurons by IH_30_^18^. We found that MnTMPyP administration was also effective in mitigating the negative regulation of both IH_10_ Tbr2^+^ INPs and the effects of IH_LATE_ on the generation of adult-born neurons. Although our results do not explicitly discriminate the contribution of the IH-mediated reduction in Tbr2^+^ INPs to the IH_LATE_ phenomenon, the twenty-day duration prior to IH exposure in the IH_LATE_ protocol permitted labeled neuroprogenitors to develop normally in room air. The period of development in room air when using the IH_LATE_ protocol extends beyond the documented period where Tbr2 expression is last observed in birth dated neural progenitor cells of the SGZ^34,66^. Thus, our findings support the notion that IH negatively regulates adult neurogenesis during the first and second critical periods. Our data also suggest that the pro-oxidant condition produced by IH has a central role for IH in negatively regulating the process during both the first and second critical periods of adult neurogenesis.

In conclusion, our novel findings reveal mechanistic complexities into the impact of IH on hippocampal adult neurogenesis. While the IH-dependent pro-oxidant state appears to negatively regulate both critical periods, cell-autonomous HIF1a signaling during the first critical period of adult neurogenesis appears to orchestrate adaptations that enhance the neuronal generation following termination of IH. Our analyses also indicate such signaling mitigates, but does not override the negative regulation of adult neurogenesis when IH is experienced over the entire period of development from NSCs to neuronal fate. Thus, the influence of IH over hippocampal adult neurogenesis appears to be determined by the relative weight of stage-related phenomena mediated by IH. Our work may be relevant for understanding how untreated sleep apnea attenuates postnatal neurogenesis and how this process may respond following treatment of the condition.

## Methods

### Study approval

In accordance with National Institutes of Health guidelines, all animal protocols were performed with the approval of the Institute of Animal Care and Use Committee at The University of Chicago.

### Experimental animals

Mice from both sexes (beginning at P30 to 35 days) were used in all experiments. Animals were given ad libitum access to food and water and were housed in ALAAC-approved facilities on a 12 h/12 h light–dark cycle. All mice were maintained on a C57BL/6 background. Nestin-CreERT2/Ai27D and Nestin-CreERT2/Ai27D/HIF1a^WT/flox^ (Nestin-CreERT2 mice from (Imayoshi et al.)^67^; Ai27D mice, The Jackson Laboratory, RRID: IMSR_JAX:012,567; and HIF1a^flox/flox^ mice, The Jackson Laboratory, RRID: IMSR_JAX:007,561) mice were used for birth dated experiments. Genotyping was performed using a commercial service (Transnetyx, Cordova, TN). No sex- based differences were observed unless otherwise described in the text.

### IH exposure

Mice were exposed to intermittent hypoxia (IH) as previously described^68^. Animals were housed in home cages and the entire home cage was placed in a custom IH chamber (0.62 × 0.55 × 0.29 m^3^) during IH exposure. Four different paradigms of IH were used in this study: ten days of IH exposure without birth dating (IH_10_); birth dating followed by ten days of IH and succeeded by twenty days in room air (IH_EARLY_, Fig. 2A Top); birth dating followed by twenty days in room air and succeeded by ten days of IH (IH_LATE_, Fig. 2A Middle); or birth dating followed by thirty days of IH (IH_30_, Fig. 2A Bottom). IH_EARLY_ was used to determine the effects of IH on cell types found in the first critical period, while IH_LATE_ was used to determine the effects of IH on cell types found in the second critical period. The IH paradigm was executed during the light cycle and lasted 7.5 ± 1.0 h per day (i.e., approximately 80 intermittent hypoxia cycles/ day) per day of IH. A single hypoxic cycle consisted of flowing 100% N_2_ into the chamber for approximately 60 s. This created a hypoxic environment where the nadir O_2_ chamber reached 4.5 ± 1.5% for 7 to 10 s and was immediately followed by an air break (21% O_2_; 300 s). Continuous vacuum was created within the chamber to balance the pressure between in and out flow of gases and ambient O_2_ was continuously monitored by sampling the air in the chamber. Control animals in their home cages were placed in similar chambers with continuous vacuum with steady room air. Following the end of each daily IH exposure, all cages were transported back into housing racks during the dark cycle.

### Antioxidant administration

In a subset of animals, administration of manganese(III) tetrakis(1-methyl-4-pyridyl)porphyrin (MnTMPYP), a superoxide anion pharmacological scavenger, (Enzo Life Sciences; https://www.enzolifesciences.com/ALX-430-070/mntmpyp-.-pentachloride/; 5 mg/kg) was intraperitoneally injected into subjects prior to the start of each daily IH exposure. A separate IH_10_ group was injected with a similar volume of saline to control for the effect of the injection.

### Tissue processing and histological analyses

Mice were anesthetized according to IACUC-approved protocols with isoflurane and transcardially perfused using saline and 4% paraformaldehyde. Brains were dissected and post-fixed in 4% paraformaldehyde overnight. Brains were cryoprotected in 30% sucrose for a minimum of 2 days and frozen in blocks using optimal cutting temperature medium. Blocks were stored at − 80 °C until sectioned and stained. Blocks containing a single hemisphere from each animal were coronally sectioned at a thickness of 40 μm on a Leica cryostat, and stored in a cryoprotectant solution of primarily glycerol at − 20 °C until used.

Every 12th section was sampled, ensuring each animal in the study had at least three usable sections through the septal region of the dentate gyrus that contained both the suprapyramidal and infrapyramidal blades. Immunohistochemistry was performed on floating sections using fluorescent dye-conjugated secondary antibodies, as previously described^69,70^. All protocols included an overnight, approximately 18 h, exposure to the primary antibodies used and a two-hour exposure to fluorescently conjugated secondary antibodies. Primary antibodies used in the present study are presented in Table 1. Sox2, Tbr2, S100B, Ki67, activated caspase-3, and HIF1a staining required additional retrieval using 0.1% citrate buffer solution prior to incubation of the primary antibodies^70^.

**Table 1:** Antibodies employed in this study.

Primary antibody	Species	Dilution	Source	RRID
Sox2^a^	Mouse	1:250	R&D Systems; MAB2018	AB_358009
Tbr2^a^	Rat	1:400	eBioscience; 14–4875-80	AB_11043546
S100B^a^	Rabbit	1:3000	Abcam; ab41548	AB_956280
RFP	Rabbit	1:400	Abcam; ab62341	AB_945213
Ki67	Rabbit	1:200	Abcam; ab15580	AB_443209
Ki67	Mouse	1:200	BD Pharmingen, 550,609	AB_393778
HIF1a^a^	Rabbit	1:250	Novus Biologicals; NB100-479	AB_10000633
Activated caspase-3^a^	Rabbit	1:400	Millipore	AB_3623

^a^Required antigen retrieval.

### Pulse labeling

Pulse labeling experiments were performed using Nestin-CreERT2;Ai27D and Nestin-CreERT2;Ai27D;HIF1a ^WT/flox^ mice (Postnatal day 29 to 34) to birth date and fate map a discrete cohort of neural progenitor cells. As previously described, expression of the Ai27D reporter was achieved in Nestin-expressing cells using 180 mg/kg tamoxifen (Fisher Scientific; cat: 54965-24-1; https://www.fishersci.com/shop/products/tamoxifen-citrate-98-acros-organics-2/p-194883) dissolved in corn oil, and delivered via intraperitoneal injection)^18^. Tamoxifen was administered up to four times separated by minimum of 8 h prior to exposure to IH. Brains were harvested for immunohistochemical study 10 to 31 days following the final day of tamoxifen administration. Birth-dated cells expressed the reporter molecule red fluorescent protein, td-tomato (RFP), fused to membrane-bound channelrhodopsin2. Immunostaining for RFP was used to identify birth-dated cells.

For counts of proliferating cells and of neural progenitor cells, counts were conducted in the SGZ. The SGZ region of interest was identified as the two to three cell thick layer at the border of the granular cell layer and hilus^71^. The granular cell layer was determined using DAPI staining and the hilus was defined as the area between the two dentate blades.

### Immunohistochemistry quantitation

Z-stack images were captured within the entire section of the dentate gyrus using a ×20, 1.3 N.A. air objective) on a Leica SP5 Tandem Scanner Spectral 2-Photon confocal microscope (Leica Microsystems, Inc., Buffalo Grove, IL) in The University of Chicago’s Imaging Core. Multiple images were required to capture the complete dentate gyrus within each 40 μm coronal slice. Images were quantified using Image J software (ImageJ, RRID:SCR_003070). Cells in the section’s entire region of interest, across multiple images, were counted using a modified macro originally provided by the UChicago Imaging Core. Cells intersecting the top-plane of each image were excluded. Cells per DG were estimated and expressed using Cavalieri’s principle: raw counts for all imaged sections were multiplied by the distance (d) between sections sampled.

### TBARS assay

Whole cell protein lysates were isolated from entire hippocampal tissues using RIPA buffer (Thermo Fisher, Waltham, CA, USA) in the presence of protease and phosphatase inhibitors (Thermo Fisher, Waltham, CA, USA) in cold ice. Protein lysates were immediately processed and stored at − 80 °C until used. The amount of lipid-peroxidation was determined using a TBARS Assay Kit (Cayman Chemical, Cat#10009055), per manufacturer instructions and absorbance was measured at a wavelength between 530–540 nm using a plate reader. The analysis to determine MDA values was made according to manufacturer instructions.

### Diagrams, experimental design, and statistical analyses

The neurogenesis diagrams shown in Figs. 1 and 2A were created using BioRender.com. All “n” are expressed as the total number of animals except otherwise noted. Statistics were performed using Prism 6 (GraphPad Software, Inc.; RRID:SCR_015807). Comparisons between two groups were conducted using unpaired two-tailed t-tests with Welch’s correction unless otherwise stated. Comparisons between multiple groups were conducted using a one-way ANOVA with a post-hoc Dunnett’s multiple comparisons test. The equality of variances between two groups was determined with an F test. Data are presented as individual data points overlaid on the mean $\pm$ S.E.M. Significance was defined as P < 0.05.

## Supplementary Information

Supplementary Information
