# Supplementary material for: Stage-dependent effects of intermittent hypoxia influence the outcome of hippocampal adult neurogenesis
Source: Sci Rep. 2021 Mar 16;11:6005. doi: 10.1038/s41598-021-85357-5 (PMC7966401; doi:10.1038/s41598-021-85357-5)
Supplement: Supplementary file 1 — Supplementary Information [file 41598_2021_85357_MOESM1_ESM.pdf]

Title: Stage-dependent effects of intermittent hypoxia influence the outcome of hippocampal adult neurogenesis

Authors: Maggie A. Khuu, Thara Nallamothu, Carolina I. Castro-Rivera, Alejandra Arias-Cavieres, Caroline C Szujewski, and Alfredo J. Garcia III

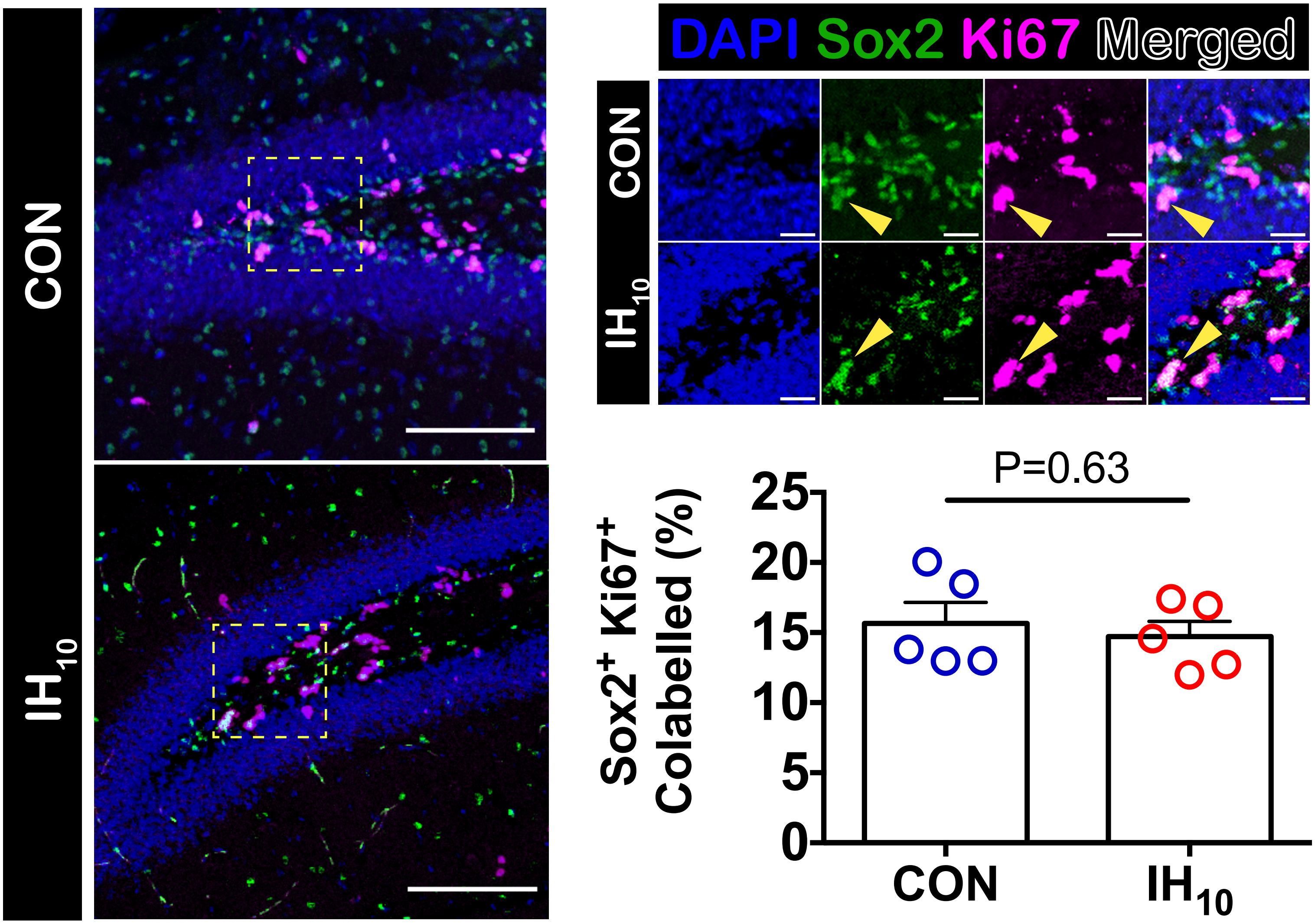

Supplementary Figure S1: Proliferation of Sox2^{+} cells is unchanged following IH$_{10}$.

The proportion of Sox2^{+}/Ki67^{+} NSCs were similar between CON and IH$_{10}$ (CON: n=5, IH$_{10}$: n=5). (left) Representative images for CON and IH$_{10}$ (left). Scale bars are 100mm. (right) Magnified images of regions highlighted by yellow dashed boxes. Scale bars for inset images are 20mm. No difference between Sox2^{+}/Ki67^{+} cells were observed between CON and IH$_{10}$.

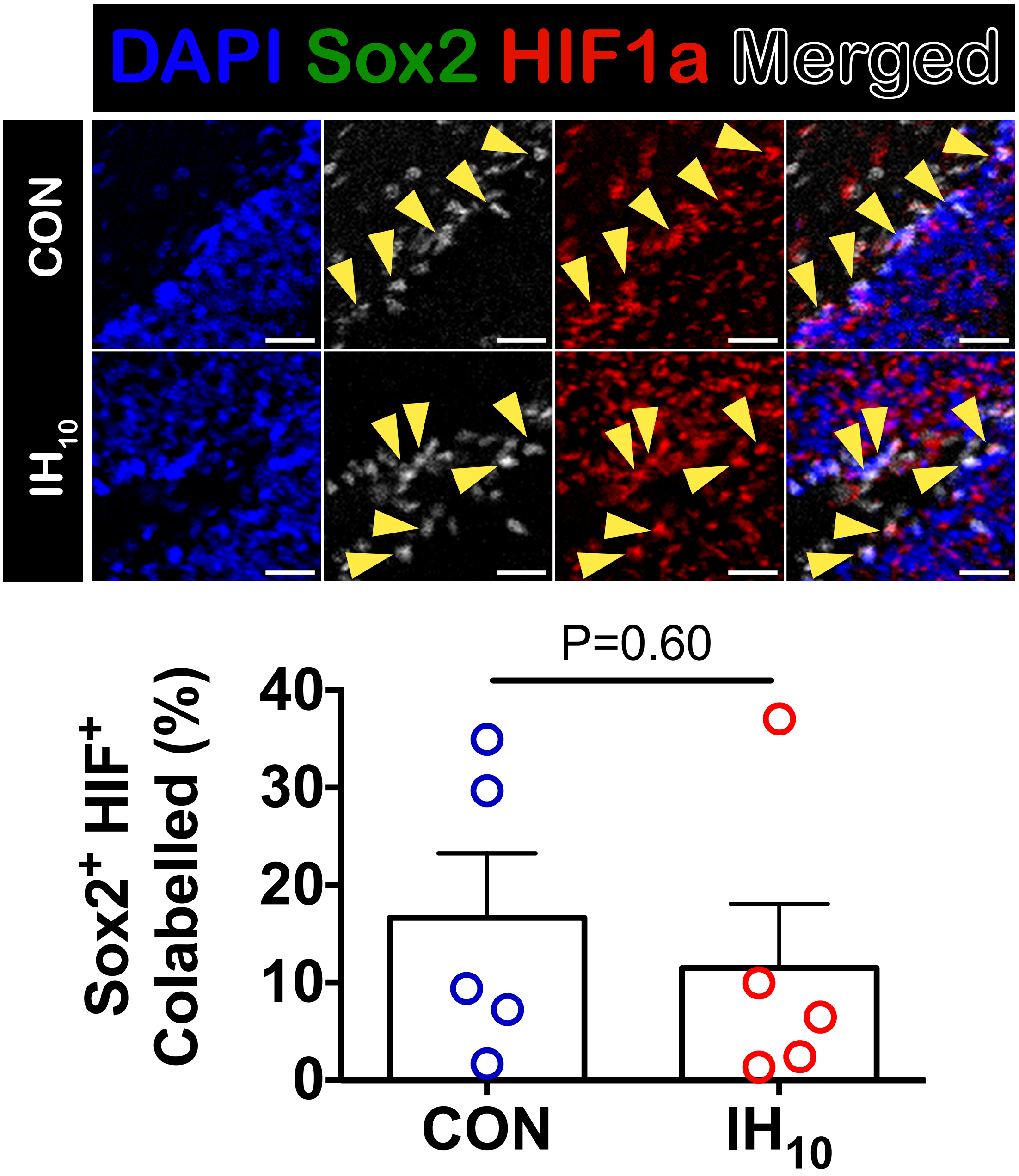

Supplementary Figure S2: No change in HIF1a expression in neural stem cell population

Representative images of HIF1a+ and Sox2+ cells in CON and IH_{10}. Yellow triangles indicate examples of co-labeled cells for CON and IH_{10}. Scale bars are 20mm. No differences in the proportion of HIF1a+/Sox2+ cells were observed following IH_{10} (CON: n=5, IH_{10}, n=5)

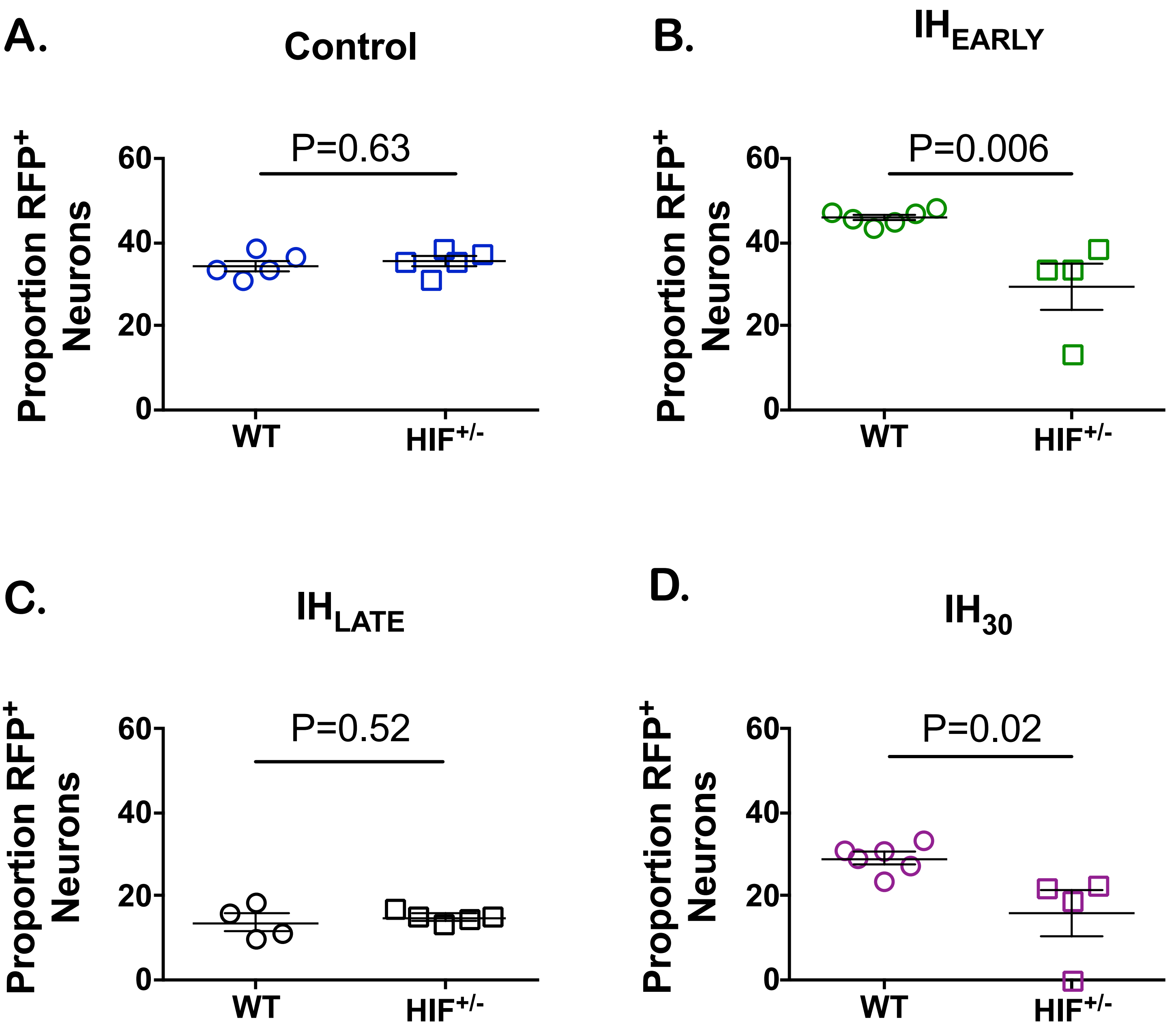

Supplementary Figure S3: Comparison of different IH conditions between genotypes.

Proportion of birth dated neurons from WT and $HIF^{+/-}$ groups (originally from Figure 2C and Figure 7B) were reanalyzed for A) Control; the number of birth dated neurons after 30 days without exposure to IH, B) IH_{EARLY}; the number of birth dated neurons in animals that received IH for ten days followed by 20 days of recovery, C) IH_{LATE}; the number of birth dated neurons in animals that waited for 20 days post tamoxifen injection before receiving ten days of IH, and D) IH_{30}; the number of birth dated neurons in animals that received IH for 30 days. All statistics were run using an unpaired t-test.
